# Information Geometry, Fluctuations, Non-Equilibrium Thermodynamics, and Geodesics in Complex Systems

**DOI:** 10.3390/e23111393

**Published:** 2021-10-24

**Authors:** Eun-jin Kim

**Affiliations:** Center for Fluid and Complex Systems, Coventry University, Priory St, Coventry CV1 5FB, UK; ejk92122@gmail.com

**Keywords:** information geometry, entropy, information rate, information length, fluctuations, Langevin equations, Fokker-planck equation, time-dependent probability density functions, self-organization

## Abstract

Information theory provides an interdisciplinary method to understand important phenomena in many research fields ranging from astrophysical and laboratory fluids/plasmas to biological systems. In particular, information geometric theory enables us to envision the evolution of non-equilibrium processes in terms of a (dimensionless) distance by quantifying how information unfolds over time as a probability density function (PDF) evolves in time. Here, we discuss some recent developments in information geometric theory focusing on time-dependent *dynamic* aspects of non-equilibrium processes (e.g., time-varying mean value, time-varying variance, or temperature, etc.) and their thermodynamic and physical/biological implications. We compare different distances between two given PDFs and highlight the importance of a path-dependent distance for a time-dependent PDF. We then discuss the role of the information rate $\Gamma =\frac{dL}{dt}$ and relative entropy in non-equilibrium thermodynamic relations (entropy production rate, heat flux, dissipated work, non-equilibrium free energy, etc.), and various inequalities among them. Here, L is the information length representing the total number of statistically distinguishable states a PDF evolves through over time. We explore the implications of a geodesic solution in information geometry for self-organization and control.

## 1. Introduction

Information geometry refers to the application of the techniques of differential geometry to probability and statistics. Specifically, it uses differential geometry to define the metric tensor that endows the statistical space (consisting of probabilities) with the notion of distance [1,2,3,4,5,6,7,8,9,10,11,12,13,14,15,16,17,18,19,20,21,22,23,24,25,26,27,28,29,30,31]. While seemingly too abstract, it permits us to measure quantitative differences among different probabilities. It then makes it possible to link a stochastic process, complexity, and geometry, which is particularly useful in classifying a growing number of data from different research areas (e.g., from astrophysical and laboratory systems to biosystems). Furthermore, it can be used to obtain desired outcomes [6,7,8,9,10,15] or to understand statistical complexity [4].

For instance, the Wasserstein metric [6,7,8,9,10] was widely used in the optimal transport problem where the main interest is to minimize transport cost which is a quadratic function of the distance between two locations. It satisfies the Fokker-Planck equation for gradient flow which minimizes the entropy/energy functional [7]. For Gaussian distributions, the Wasserstein metric space consists of *physical distances* – Euclidean and positive symmetric matrices for the mean and variance, respectively (e.g., see [8]).

In comparsion, the Fisher (Fisher-Rao) information [32] can be used to define a *dimensionless* distance in statistical manifolds [33,34]. For instance, the statistical distance $ds_{}$ represents the number of indistinguishable states as [5,33]

(1) $${\left(ds\right)}_{}^{2}\equiv \sum_{j}\frac{dp_{j}^{2}}{p_{j}}=\sum_{j}p_{j}{\left(d\ln p_{j}\right)}^{2}=\sum_{j,\alpha ,\beta }p_{j}\frac{\partial \ln p_{j}}{\partial \lambda^{\alpha }}\frac{\partial \ln p_{j}}{\partial \lambda^{\beta }}d\lambda^{\alpha }d\lambda^{\beta }=\sum_{\alpha ,\beta }d\lambda^{\alpha }g_{\alpha \beta }d\lambda^{\beta }.$$

Here the Fisher information metric $g_{\alpha \beta }=\langle \frac{\partial \ln p_{j}}{\partial \lambda^{\alpha }}\frac{\partial \ln p_{j}}{\partial \lambda^{\beta }}\rangle =\sum_{j}p_{j}\frac{\partial \ln p_{j}}{\partial \lambda^{\alpha }}\frac{\partial \ln p_{j}}{\partial \lambda^{\beta }}$ provides natural (Riemannian) distinguishability metric on the space of probability distributions. $\lambda^{\alpha }$’s are the parameters of the probability $p_{j}$ and the angular brackets represent the ensemble average over $p_{j}$. Note that Equation (1) is given for a discrete probability $p_{j}$. For a continuous Probability Density Function (PDF) p(x) for a variable *x*, Equation (1) becomes ${\left(ds\right)}^{2}=\int dxp\left(x\right){\left[d\ln\left(p(x\right)\right]}^{2}=\sum_{\alpha ,\beta }d\lambda^{\alpha }g_{\alpha \beta }d\lambda^{\beta }$ where $g_{\alpha \beta }=\int dx\;p\left(x\right)\frac{\partial \ln\left(p(x)\right)}{\partial \lambda^{\alpha }}\frac{\partial \ln\left(p(x)\right)}{\partial \lambda^{\beta }}$.

For Gaussian processes, the Fisher metric is inversely proportional to the covariance matrices of fluctuations in the systems. Thus, in thermodynamic equilibrium, strong fluctuations lead to a strong correlation and a shorter distance between the neighboring states [34,35]. Alternatively, fluctuations determine the uncertainty in measurements, providing the resolution (the distance unit) that normalizes the distance between different thermodynamic states.

To appreciate the meaning of fluctuation-based metric, let us consider the (equilibrium) Maxwell-Boltzmann distribution $p\left(E_{j}\right)=\beta e^{-\beta E_{j}}$ for the energy state $E_{j}$

(2) $$\frac{p(E_{i})}{p(E_{j})}=e^{-\beta (E_{i}-E_{j})}.$$

Here $\beta =1/k_{B}T$ is the inverse temperature; $k_{B}$ is the Boltzmann constant; *T* is the temperature of the heat bath. In Equation (2), the thermal energy $k_{B}T=\langle E\rangle$ of the heat bath (the width/uncertainty of the probability) provides the resolution to differentiate different states $\Delta E=E_{i}-E_{j}$. The smaller is the resolution (temperature), the more distinguishable states (more accessible information in the system) there are. It agrees with the expectation that a PDF gradient (the Fisher-information) increases with information [32].

This concept has been generalized to non-equilibrium systems [36,37,38,39,40,41,42,43], including the utilization for controlling systems to minimize entropy production [38,40,42], the measurement of the statistical distance in experiments to validate theoretical predictions [41], etc. However, some of these works rely on the equilibrium distribution Equation (2) that is valid only in or near equilibrium while many important phenomena in nature and laboratories are often far from equilibrium with strong fluctuations, variability, heterogeneity, or stochasticity [44,45,46,47,48,49,50,51,52]. Far from equilibrium, there is no (infinite-capacity) heat bath that can maintain the system at a certain temperature, or constant fluctuation level. One of the important questions far from equilibrium is indeed to understand how fluctuation level $\beta {\left(t\right)}^{-1}$ changes with time. Furthermore, PDFs no longer follow the Maxwell-Boltzmann nor Gaussian distributions and can involve the contribution from (rare) events of large amplitude fluctuations [53,54,55,56,57,58,59,60,61,62]. Therefore, the full knowledge of time-varying PDFs and the application of information geometry to such PDFs have become of considerable interest.

Furthermore, while in equilibrium [63,64], information theoretical measures (e.g., Shannon information entropy) can be given thermodynamic meanings (e.g., heat), in non-equilibrium such interpretations are not always possible and equilibrium thermodynamic rules can break down locally (e.g., see [65,66] and references therein). Much progress on these issues has been made by different authors (e.g., [65,66,67,68,69,70,71,72,73,74,75,76,77,78,79,80,81,82]) through the development of information theory, stochastic thermodynamics, and non-equilibrium fluctuation theorems with the help of the Fisher information [32], relative entropy [83], mutual information [84,85], etc. Exemplary works include the Landauer’s principle which links information loss to the ability to extract work [86,87]; the resolution of Maxwell’s demon paradox [88]; black hole thermodynamics [89,90]; various thermodynamic inequality/uncertainty relations [65,68,91,92,93,94,95,96,97]; and linking different research areas (e.g., non-equilibrium processes to quantum mechanics [98,99,100], physics to biology [101]).

The paper aims to discuss some recent developments in the information geometric theory of non-equilibrium processes. Since this would undoubtedly span a broad range of topics, this paper will have to be selective and will focus on elucidating the *dynamic* aspect of non-equilibrium processes and thermodynamic and physical/biological implications. Throughout the paper, we highlight that time-varying measures (esp. variance) introduces extra complication in various relations, in particular, between the information geometric measure and entropy production rate. We make the efforts to make this paper self-contained (e.g., by including the derivations of some well-known results) wherever possible.

The remainder of this paper is organized as follows. Section 2 discusses different distances between two PDFs and the generalization for a time-dependent non-equilibrium PDF. Section 3 compares the distancs from Section 2. Section 4 discusses key thermodynamic relations that are useful for non-equilibrium processes. Section 5 establishes relations between information geometric quantities (in Section 2) and thermodynamics (in Section 4). In Section 6, we discuss the concept of a geodesic in information geometry and its implications for self-organization or designing optimal protocols for control. Conclusions are provided in Section 7.

## 2. Distances/Metrics

This section discusses the distance defined between two probabilities (Section 2.1) and along the evolution path of a time-dependent probability (Section 2.2). Examples and comparisons of these distances are provided in Section 3.1. For illustration, we use a PDF p(x,t) of a stochastic variable *x* and differential entropy S=−∫dxp(x,t)ln(p(x,t)) by using the unit $k_{B}=1$.

### 2.1. Distance between Two PDFs

We consider the distance between two PDFs $p_{1}=p\left(x,t_{1}\right)$ and $p_{2}=p\left(x,t_{2}\right)$ of a stochastic variable *x* at two times $t_{1}$ and $t_{2}$, respectively where $t_{1}=t_{2}$ or $t_{1}\neq t_{2}$ in general.

#### 2.1.1. Wootters’ Distance

The Wootters’ distance [5,33] is defined in quantum mechanics by the shortest distance between the two $p_{1}$ and $p_{2}$ that have the wave functions $\psi_{1}$ and $\psi_{2}$ ($p_{1}={\left|\psi_{1}\right|}^{2}$ and $p_{2}={\left|\psi_{2}\right|}^{2}$), respectively. Specifically, for given $p_{1}$ and $p_{2}$, the distance $s(p_{1},p_{2})$ between $p_{1}$ and $p_{2}$ can be parameterized by infinitely many different paths between $p_{1}$ and $p_{2}$. Letting *z* be the affine parameter of a path, we have

(3) $$s\left(p_{1},p_{2}\right)=\int_{1}^{2}ds=\int dz\frac{ds(z)}{dz}=\int dz\sqrt{\sum_{\alpha ,\beta }\frac{d\lambda^{\alpha }}{dz}g_{\alpha \beta }\frac{d\lambda^{\beta }}{dz}},$$

where ds is given in Equation (1). Among all possible paths, the minimum of $s(p_{1},p_{2}$) is obtained for a particular path that optimizes the quantum distinguishability; the (Hilbert-space) angle between the two wave functions provides such minimum distance as

(4) $$\begin{array}{ccc} W[p_{1},p_{2}] & = & \cos^{-1}\left[\int dx\;{\left[p\left(x,t_{1}\right)\right]}^{\frac{1}{2}}{\left[p\left(x,t_{2}\right)\right]}^{\frac{1}{2}}\right]. \end{array}$$

Equation (4) is for a pure state and has been generalized to mixed states (e.g., see [37,102] and references therein). Note that the Wootters’ distance is related to the Hellinger distance [43].

#### 2.1.2. Kullback-Leibler (K-L) Divergence/Relative Entropy

Kullback-Leibler (K-L) divergence between the two PDFs [83], also called relative entropy, is defined by

(5) $$\begin{array}{ccc} K(p_{1}|p_{2}) & = & \int dx\;p\left(x,t_{1}\right)\ln\left(\frac{p(x,t_{1})}{p(x,t_{2})}\right). \end{array}$$

Relative entropy quantifies the difference between a PDF $p_{1}$ and another PDF $p_{2}$. It takes the minimum zero value for identical two PDFs $p_{1}=p_{2}$ and becomes large as $p_{1}$ and $p_{2}$ become more different. However, as it is defined in Equation (5), it is not symmetric between $p_{1}$ and $p_{2}$ and does not satisfy the triangle inequality. It is thus not a metric in a strict sense.

#### 2.1.3. Jensen Divergence

The Jensen divergence (also called Jensen distance) is the symmetrized Kullback–Leibler divergence defined by

(6) $$\begin{array}{ccc} J(p_{1}|p_{2}) & = & \frac{1}{2}\left[K\left(p_{1}|p_{2}\right)+K\left(p_{2}|p_{1}\right))\right]. \end{array}$$

While the square root of the Jensen-Shannon divergence $\sqrt{J(p_{1}|p_{2})}$ is a metric [4,103], $J(p_{1}|p_{2})$ itself has also been used in examining statistical complexity (e.g., see [43,104,105]).

#### 2.1.4. Euclidean Norm

In analysis of big data, the Euclidean norm [5,106] is used, which is defined by

(7) $$|p_{1}-p_{2}{|}^{2}=\int dx\;{\left[p\left(x,t_{1}\right)-p\left(x,t_{2}\right)\right]}^{2}.$$

While Equation (7) has a direct analogy to the physical distance, it has a limitation in measuring statistical complexity due to the neglect of the stochastic nature [5]. For instance, the Wootters’ distance in Equation (4) was shown to work better than Equation (7) in capturing complexity in the logistic map [5].

### 2.2. Distance along the Path

Equations (4)–(7) can be used to define the distance between the two given PDFs $p(x,t_{1})$ and $p(x,t_{2})$ at times $t_{1}$ and $t_{2}$ ($t_{2}>t_{1}$). However, p(x,t) at the intermediate time $t=(t_{1},t_{2})$ can take an infinite number of different values depending on the exact path that a system takes between $p(x,t_{1})$ and $p(x,t_{2})$. One example would be (i) $p\left(x,t_{1}\right)=p\left(x,t_{2}\right)=p\left(x,t\right)$ for all $t=(t_{1},t_{2})$ and *x*, in comparison with (ii) $p\left(x,t_{1}\right)=p\left(x,t_{2}\right)$ but $p\left(x,t\right)\neq p\left(x,t_{1}\right)$ and $p\left(x,t\right)\neq p\left(x,t_{2}\right)$. What is necessary is a *path-dependent* distance that depends on the exact evolution and the form of p(x,t) for $t=(t_{1},t_{2})$.

#### 2.2.1. Information Rate

Calculating a path-dependent *distance* for a time-dependent PDF p(x,t) requires the generalization of the distance in Section 2.1. To this end, we consider two (temporally) adjacent PDFs along the trajectory, say, p(x,t) and p(x,t+dt) and calculate the (infinitesimal) relative entropy between them in the limit dt→0 to the leading order in O(dt):

(8) $$\begin{array}{ccc} & & \lim_{dt\to 0}\frac{1}{{\left(dt\right)}^{2}}K\left[p\left(x,t+dt\right)|p\left(x,t\right)\right]=\lim_{dt\to 0}\frac{1}{{\left(dt\right)}^{2}}K\left[p\left(x,t\right)|p\left(x,t+dt\right)\right]\\ & & =\lim_{dt\to 0}\frac{1}{{\left(dt\right)}^{2}}J\left[p\left(x,t+dt\right)|p\left(x,t\right)\right]=\frac{1}{2}\int dxp\left(x,t\right){\left(\partial_{t}\ln p(x,t)\right)}^{2}\equiv \frac{1}{2}\Gamma^{2}. \end{array}$$

Here, we used $p\left(x,t+dt\right)=p\left(x,t\right)+\left(dt\right)\partial_{t}p+\frac{1}{2}{\left(dt\right)}^{2}{\left(\partial_{t}p\right)}^{2}$ + O(${\left(dt\right)}^{3}$), $\ln\left(1+r\right)=r-\frac{1}{2}r^{2}$ + O($r^{3}$) for r≪1, and $\int dx\partial_{t}p\left(x,t\right)=\int dx\partial_{tt}p\left(x,t\right)=0$ because of the total probability conservation ∫dxp(x,t)=1. Due to the symmetry of K[p(x,t+dt)|p(x,t)] to leading order O(${\left(dt\right)}^{2}$), K[p(x,t+dt)|p(x,t)]=J[p(x,t+dt)|p(x,t)] to O(${\left(dt\right)}^{2}$).

In Equation (8), the information rate Γ is defined by [15,16,17,18,19,20,21,22,23,24,25,26,27,28,29]

(9) $$\Gamma^{2}\left(t\right)=\lim_{dt\to 0}\frac{2}{{\left(dt\right)}^{2}}J\left[p\left(x,t+dt\right)|p\left(x,t\right)\right]=\int dxp\left(x,t\right){\left(\partial_{t}\ln p(x,t)\right)}^{2}=4\int dx\;{\left(\partial_{t}q\left(t\right)\right)}^{2}.$$

Here, $q=\sqrt{p}$, and Γ≥0 by definition. We note that the last term in terms of *q* in Equation (9) can be used when q=p=0. The dimensions of $\Gamma^{2}\equiv E$ and Γ are (time)${}^{-2}$ and (time)${}^{-1}$, respectively. They do not change their values under nonlinear, time-independent transformation of variables (see Appendix A). Thus, using the unit where the length is dimensionless, E and Γ can be viewed as the kinetic energy per mass and velocity, respectively. For this reason, Γ was called the velocity (e.g., in [15,17]).

Note that E can be viewed as the Fisher information [32] if time is interpreted as a parameter (e.g., [97]). However, time in classical mechanics is a passive quantity that cannot be changed by an external control. Γ is also called the entropy production rate in quantum mechanics [107]. However, as shown in Section 4.1 and Section 4.4, the relation between Γ and thermodynamic entropy production rate is more complicated (see Equation (28)).

Γ in Equation (9) is the information rate representing how quickly new information is revealed as a PDF evolves in time. Here, $\Gamma^{-1}=\tau$ is the characteristic time scale of this information change in time. To show that Γ is related to fluctuation’s smallest time scale [97], we assume that $\lambda^{\alpha }$’s are the estimators (parameters) of a p(x,t) and use the Cramér-Rao bound on the Fisher information $g_{\alpha \beta }=\int dxp\left(x,t\right)\partial_{\lambda^{\alpha }}\left[\ln p(x,t)\right]\partial_{\lambda^{\beta }}\left[\ln p(x,t)\right]\geq C_{\alpha \beta }^{-1}$ where $C^{\alpha \beta }\equiv \langle \delta \lambda^{\alpha }\delta \lambda^{\beta }\rangle$ is the covariance matrix (e.g., see [32]); $\delta \lambda^{\alpha }=\lambda^{\alpha }-\langle \lambda^{\alpha }\rangle$ denotes fluctuation. Using $\frac{d\ln p}{dt}=\frac{\partial \ln p}{\partial \lambda^{\alpha }}\frac{d\lambda^{\alpha }}{dt}$ then leads to

(10) $$\begin{array}{ccc} \Gamma^{2} & = & \sum_{\alpha \beta }\frac{d\lambda^{\alpha }}{dt}g_{\alpha \beta }\frac{d\lambda^{\beta }}{dt}\geq \sum_{\alpha \beta }\frac{d\lambda^{\alpha }}{dt}C_{\alpha \beta }^{-1}\frac{d\lambda^{\beta }}{dt}. \end{array}$$

For the diagonal $g_{\alpha \beta }=g_{\alpha }\delta_{\alpha \beta }$, Equation (10) is simplified as

(11) $$\begin{array}{ccc} \sum_{\alpha }\frac{1}{\langle {\left(\delta \lambda^{\alpha }\right)}^{2}\rangle }{\left(\frac{d\lambda^{\alpha }}{dt}\right)}^{2} & \leq & \Gamma^{2}. \end{array}$$

Equation (11) shows how the RMS fluctuation-normalized rate at which the parameter $\lambda^{\alpha }$ can change is bounded above by Γ. If there is only α=1 ($\lambda^{\alpha }=\lambda \delta_{\alpha ,1}$), Equation (11) is further simplified:

(12) $$\begin{array}{cc} \frac{1}{\sqrt{\langle {\left(\delta \lambda \right)}^{2}\rangle }}\left|\frac{d\lambda }{dt}\right| & \leq \Gamma , \end{array}$$

clearly showing that λ normalized by its RMS fluctuations cannot change faster than the information rate.

Finally, it is worth highlighting that Equation (9) is general and can be used even when the parameters $\lambda^{\alpha }$’s and $g_{\alpha \beta }$ in $\Gamma^{2}$ in Equation (10) are unknown. Examples include the cases where PDFs are empirically inferred from experimental/observational data. Readers are referred to Refs. [21,23,28] for examples. It is only the special case where we have a complete set of parameters $\lambda^{\alpha }$’s of a PDF that we can express Γ using the Fisher information as in Equation (10). For instance, for a Gaussian p(x,t) that is fully described by the mean value 〈x〉 and variance $\frac{1}{2\beta }$, $\left(\lambda^{1},\lambda^{2}\right)=\left(\langle x\rangle ,\beta \right)$.

#### 2.2.2. Information Length

Since $\Gamma \propto \sqrt{J[p(x,t+dt)|p(x,t)]}$ is a metric [103] as noted in Section 2.1, Γ is also a metric. Thus, we sum Γ along the trajectory to define a finite distance. Specifically, starting with an initial PDF p(x,t=0), we integrate Γ(t) over time to obtain the dimensionless number as a function time as

(13) $$\begin{array}{ccc} L(t) & = & \int_{0}^{t}dt_{1}\Gamma \left(t_{1}\right). \end{array}$$

L is the information length [15,16,17,18,19,20,21,22,23,24,25,26,27,28,29,30,31] that quantifies the total change in information along the trajectory of p(x,t) or the total number of statistically distinguishable states it evolves through over time. [We note that different names (e.g., statistical length [108], or statistical distance [97]) were also used for L.] It is important to note that unlike the Wootters’ distance (the shortest distance among all possible paths between the two PDFs) in Equation (3) (e.g., [5]), L(t) in Equation (13) is fixed for a given time-evolving PDF p(x,t).

By definition in Equation (13), L(t=0)=0 and L(t) monotonically increases with time since Γ≥0 (e.g., see Figure A2 in [22]). L(t) takes a constant value only in a stationary state ($\Gamma =\partial_{t}p=0$). One of its important consequences is that when p(x,t) relaxes into a stationary PDF in the long time limit t→∞, Γ(t)→0 and $L\left(t\right)\to L_{\infty }$ as t→∞ where $L_{\infty }$ is a constant depending on initial conditions and parameters. This property of $L_{\infty }$ was used to understand attractor structure in a relaxation problem; specifically, Refs. [15,16,18,22,28] calculated $L_{\infty }$ for different values of the mean position $x_{0}$ of an initial PDF and examined how $L_{\infty }$ depends on $x_{0}$. Furthermore, Γ and L were shown to be useful for quantifying hysteresis in forward-backward processes [19], correlation and self-regulation among different players [23,25], and predicting the occurrence of sudden events [27] and phase transitions [23,25]. Some of these points are illustrated in Section 3.1.

## 3. Model and Comparison of Metrics

For discussing/comparing different metrics in Section 2 and statistical measures in Section 4, we use the following Langevin model [109]

(14) $$\frac{dx}{dt}=f\left(x,t\right)+\xi =-\partial_{x}V\left(x,t\right)+\xi .$$

Here, V(x,t) is, in general, a time-dependent potential which can include an internal potential and an external force; ξ is a short (delta)-correlated Gaussian noise with the following statistical property

(15) $$\begin{array}{ccc} & & \langle \xi \left(t\right)\xi \left(t^{\prime }\right)\rangle =2D\delta \left(t-t^{\prime }\right). \end{array}$$

Here, the angular brackets represent the ensemble average over the stochastic noise ξ; D≥0 is the amplitude of ξ. It is important to note that far from equilibrium, the average (e.g., 〈x(t)〉) is a function of time, in general.

The exact PDFs can be obtained for the Ornstein-Uhlenbeck (O-U) process which has $V=\frac{\gamma }{2}{\left(x-v\left(t\right)\right)}^{2}$ and f=−γ(x−v(t)) in Equation (14). Here, v(t) is a deterministic function of time. Specifically, for the initial Gaussian PDF p(x,0)

(16) $$p\left(x,0\right)=\sqrt{\frac{\beta_{0}}{\pi }}e^{-\beta_{0}{\left(x-x_{0}\right)}^{2}},$$

a time-dependent PDF remains Gaussian at all time:

(17) $$\begin{array}{ccc} p(x,t) & = & \int dx_{1}p\left(x,t;x_{1},0\right)p\left(x_{1},0\right)=\sqrt{\frac{\beta }{\pi }}e^{-\beta {\left(x-\langle x\rangle \right)}^{2}}, \end{array}$$

(18) $$\begin{array}{ccc} \frac{1}{2\beta (t)} & = & \frac{e^{-2\gamma t}}{2\beta_{0}}+\frac{D(1-e^{-2\gamma t})}{\gamma }, \end{array}$$

(19) $$\begin{array}{ccc} \langle x(t)\rangle & = & x_{0}e^{-\gamma t}+\gamma \int_{0}^{t}dt_{1}\;e^{-\gamma (t-t_{1})}v\left(t_{1}\right). \end{array}$$

In Equations (16)–(19), $x_{0}=\langle x\left(t=0\right)\rangle$, $\beta_{0}=\beta \left(t=0\right)$, and $\langle {\left(\delta x\right)}^{2}\rangle =\frac{1}{2\beta }=\sigma^{2}$. Here, β, σ and $\sigma^{2}$ are the inverse temperature, standard deviation, and variance, respectively; $\beta_{0}$ and $x_{0}$ are the values of β and 〈x〉, respectively, at t=0. Equation (18) shows that as t→∞, $\beta \left(t\to \infty \right)=\frac{\gamma }{2D}$. Note that we use both β and σ here to clarify the connections to the previous works [15,17,22,26,27,28].

### 3.1. Geometric Structure of Equilibrium/Attractors

To elucidate the main difference between the distances in Equations (4)–(6) and Equation (13), we consider the relaxation problem by assuming v(t)=0. In the following, we compare the distance between p(x,0) and p(x,t→∞) by using $p_{1}\left(x,t_{1}\right)=p\left(x,0\right)$ and $p_{2}\left(x,t_{2}\right)=p\left(x,t\to \infty \right)$ in Equations (4)–(6) and Equation (13). Analytical expressions for these distances are given in [22].

**Figure 1:**
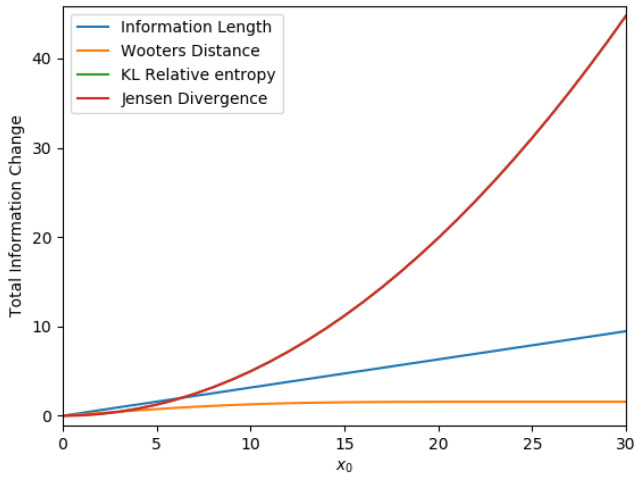
The distance against $x_{0}$ between p(x,0) and p(x,t→∞) for the O-U process. (Figure 1 in [22]).

Each curve in Figure 1 shows how each distance depends on the initial mean position $x_{0}$. The four different curves are for $L_{\infty }$ (in blue), Wootters’ distance (in orange), K-L relative entropy (in green), and Jensen divergence (in red), respectively. The relative entropy and Jensen divergence exhibit similar behavior, the red and green color curves being superimposed on each other. Of note is a linear relation between $L_{\infty }$ and $x_{0}$ in Figure 1. Such linear relation is not seen in other distances. This means that the information length is a unique measure that manifests a linear geometry around its equilibrium point in a linear Gaussian process [28,30]. Note that for a nonlinear force *f*, $L_{\infty }$ has a power-law relation with $x_{0}$ for a sufficiently large $x_{0}$ [18,28]. These contrast with the behaviour in a chaotic system [16,28] where $L_{\infty }$ depends sensitively on the initial condition and abruptly changes with $x_{0}$. Thus, the information length provides a useful tool to geometrically understand attractor structures in relaxation problems.

### 3.2. Correlation between Two Interacting Components

**Figure 2:**
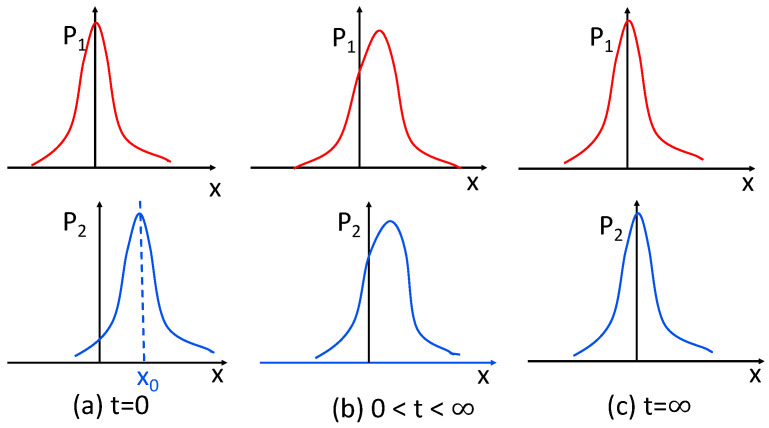
$P_{1}$ (top) and $P_{2}$ (bottom) at time t=0 in panel (**a**), t=(0,∞) in panel (**b**), and t→∞ in panel (**c**). Note that $P_{1}\left(0<t<\infty \right)\neq P_{1}\left(t=0\right)$ ($=P_{1}\left(t\to \infty \right))$.

We next show that the information length is also useful in elucidating the correlation between two interacting species such as two competing components relaxing to the same equilibrium in the long time limit. Specifically, the two interacting components with the time-dependent PDFs $P_{1}\left(x,t\right)$ and $P_{2}\left(x,t\right)$ are coupled through the Dichotomous noise [110,111] (see Appendix B) and relax into the same equilibrium Gaussian PDF $P_{1}\left(x,t\to \infty \right)=P_{2}\left(x,t\to \infty \right)=\frac{1}{2}P\left(x,t\to \infty \right)$ around x=0 in the long time limit. Here, $P\left(x,t\right)=P_{1}\left(x,t\right)+P_{2}\left(x,t\right)$ is the total PDF. For the case considered below, P(x,t) satisfies the O-U process (see Appendix B for details). We choose the initial conditions where $P_{1}\left(t=0\right)=P_{1}\left(t\to \infty \right)$ with zero initial mean value while $P_{2}\left(t=0\right)$ takes an initial mean value $x_{0}$. These are demonstrated in the cartoon figure, Figure 2a,c.

Although $P_{1}\left(t=0\right)=P_{1}\left(t\to \infty \right)$, at the intermediate time t=(0,∞), $P_{1}\left(x,t\right)$ evolves in time due to its coupling to $P_{2}$ and thus $P_{1}\left(x,t\right)\neq P_{1}\left(x,t=0\right)$, as shown in Figure 2b. Consequently, L(t) calculated from $P_{1}$ monotonically increases to its asymptotic value $L_{\infty }$ until it reaches the equilibrium (see Figure A2 in [22] for time-evolution of L from $P_{1}$ and $P_{2}$). On the other hand, $P_{2}$ with an initial mean value $x_{0}$ undergoes a different time evolution (unless $x_{0}=0$) until it reaches the equilibrium.

**Figure 3:**
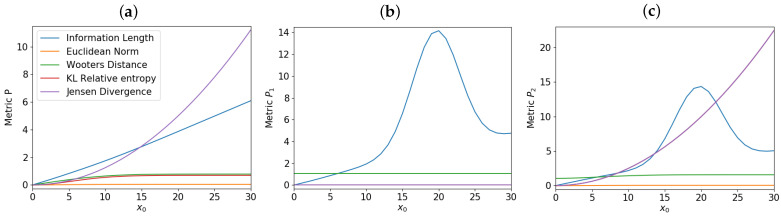
The distance between P(x,0) and P(x,t→∞) against in $x_{0}$ in (**a**); $P_{1}\left(x,0\right)$ and $P_{2}\left(x,t\to \infty \right)$ in (**b**); $P_{2}\left(x,0\right)$ and $P_{2}\left(x,t\to \infty \right)$ in (**c**). (Figure 4 in [22]).

The distances in Equations (4)–(7) and Equation (13) can be calculated from the total $P=P_{1}+P_{2}$, $P_{1}$ and $P_{2}$ for different values of $x_{0}$. Results are shown in Figure 3a–c, respectively; (a) P(x,0) and P(x,t→∞), (b) $P_{1}\left(x,0\right)$ and $P_{1}\left(x,t\to \infty \right)$, and (c) $P_{2}\left(x,0\right)$ and $P_{2}\left(x,t\to \infty \right)$, respectively. Specifically, for each value of $x_{0}$, we calculate the distances in Equations (4)–(7) and Equation (13) by using $p_{1}\left(x,t_{1}\right)=P\left(x,0\right)$ and $p_{2}\left(x,t_{2}\right)=P\left(x,t\to \infty \right)$ for Figure 3a; $p_{1}\left(x,t_{1}\right)=P_{1}\left(x,0\right)$ and $p_{2}\left(x,t_{2}\right)=P_{1}\left(x,t\to \infty \right)$ for Figure 3b; $p_{1}\left(x,t_{1}\right)=P_{2}\left(x,0\right)$ and $p_{2}\left(x,t_{2}\right)=P_{2}\left(x,t\to \infty \right)$ for Figure 3c. The same procedure above is then repeated for many other $x_{0}$’s to show how each distance depends on $x_{0}$.

For the total *P*, a linear relation between $L_{\infty }$ and $x_{0}$ is seen in Figure 3a (like in Figure 1). This linear relation is not seen in $L_{\infty }$ calculated from either $P_{1}$ or $P_{2}$ in Figure 3b or Figure 3c; a non-monotonic dependence of $L_{\infty }$ in Figure 3b,c is due to large-fluctuations and strong-correlation between $P_{1}$ and $P_{2}$ during time-evolution for large $x_{0}$. What is quite remarkable is that in contrast to other distances, $L_{\infty }$ calculated from $P_{1}$ and $P_{2}$ in Figure 3b,c exhibits a very similar dependence on $x_{0}$. It means that despite very different time-evolutions of $P_{1}$ and $P_{2}$ (see Figure 2), they undergo similar total change in information. These results suggest that strong coupling between two components can be inferred from their similar information length (see also [24,25]).

## 4. Thermodynamic Relations

To elucidate the utility of information geometric theory in understanding non-equilibrium thermodynamics, we review some of the important thermodynamic measures of irreversibility and dissipation [112] and relate them to information geometric measures Γ and K [29]. For illustration below, we use the model in Equations (14) and (15) unless stated otherwise. Corresponding to Equations (14) and (15) is the following Fokker-Planck equation [109]

(20) $$\begin{array}{ccc} \frac{\partial p(x,t)}{\partial t} & = & -\frac{\partial }{\partial x}\left[f(x,t)p(x,t)\right]+D\frac{\partial^{2}p\left(x,t\right)}{\partial x^{2}}=-\partial_{x}J\left(x,t\right), \end{array}$$

where $J=fp-D\partial_{x}p$ is the probability current.

### 4.1. Entropy Production Rate and Flow

For non-equilibrium thermodynamics, we need to consider the entropy in the system *S* and the environment $S_{m}$, and the total entropy $S_{T}=S+S_{m}$. To clarify the difference among these, we go over some derivation by using $\partial_{t}p=-\partial_{x}J$ and $J=fp-D\partial_{x}p$ to obtain

(21) $$\dot{S}=\frac{dS(x,t)}{dt}=-\int dx\partial_{t}p\ln p=\frac{dS_{T}\left(x,t\right)}{dt}-\frac{dS_{m}\left(x,t\right)}{dt},$$

where,

(22) $$\begin{array}{ccc} {\dot{S}}_{T}=\frac{dS_{T}}{dt}=\int dx\left(\frac{1}{Dp}J^{2}\right),\; & & {\dot{S}}_{m}=\frac{dS_{m}}{dt}=\int dx\left(\frac{1}{D}Jf\right). \end{array}$$

Here, we used integration by parts in *t* and *x*. ${\dot{S}}_{T}$ denotes the (total) entropy production rate, which is non-negative ${\dot{S}}_{T}\geq 0$ by definition, and serves as a measure of irreversibility [112]. The sign of ${\dot{S}}_{m}$ in Equation (22) represents the direction in which the entropy flows between the system and environment. Specifically, ${\dot{S}}_{m}>0$ (${\dot{S}}_{m}<0$) when the entropy flows from the system (environment) to the environment (system). ${\dot{S}}_{m}$ is related to the heat flux $Q=DS_{m}$ from the system to the environment. The equality ${\dot{S}}_{T}=0$ holds in an equilibrium reversible process. In this case, $\dot{S}=-{\dot{S}}_{m}=-\frac{Q}{D}$, which is the usual equilibrium thermodynamic relation. In comparison, when $\dot{S}=0$, ${\dot{S}}_{T}={\dot{S}}_{m}\geq 0$.

For the O-U process with $V=\frac{\gamma }{2}{\left(x-v\left(t\right)\right)}^{2}$ and f=−γ(x−v(t)) in Equation (14), Equations (17)–(19) and Equations (21)–(22) lead to (see [29] for details)

(23) $$\begin{array}{ccc} S(t) & = & \frac{1}{2}\left[1+\ln\frac{\pi }{\beta }\right], \end{array}$$

(24) $$\begin{array}{ccc} D\dot{S_{T}} & = & \frac{{\left(\partial_{t}\beta \right)}^{2}}{8\beta^{3}}+{\left(\partial_{t}\langle x\rangle \right)}^{2}={\left(\partial_{t}\sigma \right)}^{2}+{\left(\partial_{t}\langle x\rangle \right)}^{2}, \end{array}$$

(25) $$\begin{array}{ccc} \dot{S} & = & -\frac{\partial_{t}\beta }{2\beta }=\frac{\partial_{t}\sigma }{\sigma }, \end{array}$$

(26) $$\begin{array}{ccc} {\dot{S}}_{m} & = & {\dot{S}}_{T}-\dot{S}. \end{array}$$

Here, we used $J=\left[f+2D\beta (\delta x)\right]p=\left[-\frac{\partial_{t}\beta }{2\beta }\left(\delta x\right)+\partial_{t}\langle x\rangle \right]p$, $\partial_{x}p=-2\beta \left(\delta x\right)p$, f=−γ(x−v(t)), $\partial_{t}\langle x\rangle =\langle f\rangle$, $2D\beta -\gamma =-\frac{\partial_{t}\beta }{2\beta }$, and $\frac{\partial_{t}\beta }{\beta }=-2\frac{\partial_{t}\sigma }{\sigma }$.

In order to relate these thermodynamical quantities ${\dot{S}}_{T}$ and $\dot{S}$ above to the information rate Γ, we recall that for the O-U process [15,17,26,27,28],

(27) $$\begin{array}{ccc} E & = & \Gamma^{2}=2\beta {\left(\partial_{t}\langle x\rangle \right)}^{2}+\frac{{\left(\partial_{t}\beta \right)}^{2}}{2\beta^{2}}=\frac{1}{\sigma^{2}}\left[2{\left(\partial_{t}\sigma \right)}^{2}+{\left(\partial_{t}\langle x\rangle \right)}^{2}\right]. \end{array}$$

Equations (24) and (27) then give us

(28) $$\begin{array}{ccc} \Gamma^{2} & = & \frac{D}{\sigma^{2}}{\dot{S}}_{T}+{\dot{S}}^{2}. \end{array}$$

Interestingly, Equation (28) reveals that the entropy production rate needs be normalized by variance $\sigma^{2}$. This is because of the extensive nature of ${\dot{S}}_{T}$ unlike Γ or $\dot{S}$. That is, ${\dot{S}}_{T}$ changes its value when the variable *x* is rescaled by a scalar factor, say, α (>0) as y=αx. Furthermore, Equation (28) shows that the information rate Γ in general does not have a simple relation to the entropy production rate (c.f., [107]).

One interesting limit of Equation (28) is the case of constant β(t) with $\dot{S}=0$. In that case, Equation (24) becomes $D{\dot{S}}_{T}={\left(\partial_{t}\langle x\rangle \right)}^{2}$ while Equations (13), (27) and (28) give us

(29) $$\begin{array}{ccc} L(t) & = & \frac{\langle x(t)\rangle -\langle x(t=0)\rangle }{\sigma }, \end{array}$$

(30) $$\begin{array}{ccc} \Gamma^{2} & = & \frac{1}{\sigma^{2}}{\left(\partial_{t}\langle x\rangle \right)}^{2}=\frac{D}{\sigma^{2}}{\dot{S}}_{T}. \end{array}$$

Equation (29) simply states that L measures the total change in the mean value normalized by fluctuation level σ. Equation (30) manifests a linear relation between $\Gamma^{2}$ and ${\dot{S}}_{T}$ when $\partial_{t}\sigma =0$, as invoked in the previous works (e.g., [107]). Furthermore, a linear relation between $\Gamma^{2}$ and ${\dot{S}}_{T}$ in Equation (30) implies that minimizing the entropy production $\int^{t}dt_{1}{\dot{S}}_{T}$ along the trajectory corresponds to minimizing $\int_{0}^{t}dt_{1}\Gamma^{2}\left(t_{1}\right)$, which, in turn, is equivalent to minimizing L(t) (see Section 5 for further discussions).

Finally, to demonstrate how entropy production rate and thermal bath temperature (*D*) are linked to the speed of fluctuations c=σΓ [97], we rewrite Equation (28) as

(31) $$c=\sigma \Gamma ={\left[D{\dot{S}}_{T}+\sigma^{2}{\dot{S}}^{2}\right]}^{\frac{1}{2}}.$$

For constant variance $\dot{\beta }=0$, Equation (31) gives a simple relation $c=\sigma \Gamma =\sqrt{D{\dot{S}}_{T}}$.

### 4.2. Non-Equilibrium Thermodynamical Laws

To relate the statistical measures in Section 4.1 to thermodynamics, we let *U* (internal energy) be the average potential energy U=〈V〉 and obtain (see also [66,113] and references therein)

(32) $$\begin{array}{ccc} \frac{dU}{dt} & = & \frac{d}{dt}\langle V\rangle \equiv \dot{W}-\dot{Q}, \end{array}$$

where

(33) $$\begin{array}{ccc} \dot{W} & = & \int dx\left(\partial_{t}V\right)p=\langle \partial_{t}V\rangle , \end{array}$$

(34) $$\begin{array}{ccc} \dot{Q} & = & -\int dxV\left(\partial_{t}p\right)=\int dxJf=\langle f\dot{x}\rangle =D{\dot{S}}_{m}. \end{array}$$

The power $\dot{W}$ represents the average rate at which the work is done to the system because of time-varying potential; the average work during the time interval $\left[t_{0},t\right]$ is calculated by $W=\int_{t_{0}}^{t}dt^{\prime }\dot{W}\left(t^{\prime }\right)$. On the other hand, $\dot{Q}$ represents the rate of dissipated heat.

Equation (32) establishes the non-equilibrium thermodynamic relation $\dot{U}=\dot{W}-\dot{Q}$. Physically, it simply means that the work done to the system $\dot{W}$ increases *U* while the dissipated heat to the environment $\dot{Q}$ decreases it. Equations (21), (32), and (34) permit us to define a non-equilibrium (information) free energy F(t)=U(t)−DS(t) [92] and its time-derivative

(35) $$\begin{array}{ccc} \dot{F}=\dot{U}-D\dot{S} & = & \dot{W}-D{\dot{S}}_{T}, \end{array}$$

where $\dot{F}=\frac{dF}{dt}$ and $\dot{U}=\frac{dU}{dt}$. Since ${\dot{S}}_{T}\geq 0$, Equation (35) leads to the following inequality

(36) $$\begin{array}{ccc} D{\dot{S}}_{T} & = & \dot{W}-\dot{F}\equiv {\dot{W}}_{D}\geq 0, \end{array}$$

where the non-negative dissipated power (lost to the environment) ${\dot{W}}_{D}$ is defined. Finally, the time-integral version of Equation (36) provides the bound on the average work performed on the system as $W-\Delta F=W_{D}\geq 0$ (e.g., [68]).

### 4.3. Relative Entropy as a Measure of Irreversibility

The relative entropy has proven to be useful in understanding irreversibilities and non-equilibrium thermodynamic inequality relations [91,92,93,94,114,115,116]. In particular, the dissipated work $W_{D}=W-\Delta F$ (in Equation (36)) is related to the relative entropy between the PDFs in the forward and reverse processes

(37) $$W_{D}=DK\left[p_{F}\left(\gamma_{F}\left(t\right)\right)|p_{R}\left(\gamma_{R}\left(t\right)\right)\right].$$

(e.g., see [91,92,93,94].) Here, $p_{F}\left(\gamma_{F}\left(t\right)\right)$ and $p_{R}\left(\gamma_{R}\left(t\right)\right)$ are the PDFs for the forward and reverse processes driven by the forward $\gamma_{F}\left(t\right)$ and reverse $\gamma_{R}\left(t\right)$ protocols, respectively. Using Equation (36) in Equation (37) immediately gives

(38) $${\dot{S}}_{T}=\frac{d}{dt}K\left[p_{F}\left(\gamma_{F}\left(t\right)\right)|p_{R}\left(\gamma_{R}\left(t\right)\right)\right]\geq 0,$$

which is a proxy for irreversibility (see [115,116] for a slightly different expression of Equation (38)). It is useful to note that forward and reversal protocols are also used to establish various fluctuations theorems for different dissipative measures such as entropy production, dissipated work, etc. (see, e.g., [80] for a nice review and references therein).

However, we cannot consider forward and reversal protocols in the absence of a model control parameter that can be prescribed as a function of time. Even in this case, the relative entropy is useful in quantifying irreversibility through inequalities, and this is what we focus on in the remainder of Section 4.3.

To this end, let us consider a non-equilibrium state p(x,t) which has an instantaneous non-equilibrium stationary state $p_{s}\left(x,t\right)$ and calculate the relative entropy between the two. Here, $p_{s}\left(x,t\right)$ is a steady solution of the Fokker-Planck equation $\partial_{t}p_{s}=0$ in Equation (20) (e.g., see [29]). Specifically, one finds $p_{s}\left(x,t\right)=e^{-\frac{V\left(x,t\right)-F_{s}\left(t\right)}{D}}$ by treating the parameters to be constant (being frozen to their instantaneous values at a given time). Here, *V* and $F_{s}$ are the potential energy and the stationary free energy, respectively. For clarity, an example of $p_{s}\left(x,t\right)$ is given in Section 4.4.

The average of $\ln p_{s}\left(t\right)$ in the non-equilibrium state p(x,t) can be expressed as follows:

(39) $$\begin{array}{c} \int dxp\left(x,t\right)\ln p_{s}\left(x,t\right)=-\frac{1}{D}\int dxp\left(x,t\right)\left(V\left(x,t\right)-F_{s}\left(t\right)\right)=-\frac{1}{D}\left(U\left(t\right)-F_{s}\left(t\right)\right). \end{array}$$

Equations (35) and (39) then give us

(40) $$\begin{array}{c} F\left(t\right)-F_{s}\left(t\right)=D\int dxp\left(x,t\right)\ln\left[\frac{p(x,t)}{p_{s}\left(x,t\right)}\right]\equiv DK\left[p\left(x,t\right)|p_{s}\left(x,t\right)\right]\geq 0. \end{array}$$

Here, we used the fact the relative entropy is non-negative. Equation (40) explicitly shows that non-equilibrium free energy is bounded below by the stationary one $F\geq F_{s}$ (see also [1,92] and references therein for open Hamiltonian systems).

Equation (40) together with Equation (35) then lead to the following irreversible work $W_{irr}$ [29,92]:

(41) $$\begin{array}{c} W_{irr}\equiv W-\Delta F_{s}=D\Delta S_{T}+\Delta \left(F-F_{s}\right)=D\Delta S_{T}+D\Delta K\left[p\left(x,t\right)|p_{s}\left(x,t\right)\right]. \end{array}$$

Here, $\Delta K\left[p\left(x,t\right)|p_{s}\left(x,t\right)\right]=K\left[p\left(x,t\right)|p_{s}\left(x,t\right)\right]-K\left[p\left(x,t_{0}\right)|p_{s}\left(x,t_{0}\right)\right]$, etc. The derivation of Equation (41) for open-driven Hamiltonian systems is provided in [92] (see their Equation (38)).

On the other hand, we directly calculate the time-derivative of $K[p\left(x,t\right)|p_{s}\left(x,t\right)]$ in Equation (40) by using $p_{s}\left(x,t\right)=e^{-\frac{V\left(x,t\right)-F_{s}\left(t\right)}{D}}$, ${\dot{S}}_{T}=\dot{S}+{\dot{S}}_{m}$, $\int dxp\partial_{t}V=\dot{W}$ and $\dot{Q}=-\int dx\;\partial_{t}pV=D{\dot{S}}_{m}$, and $W_{irr}=W-\Delta F_{s}$:

(42) $$\begin{array}{ccc} & & \frac{d}{dt}K\left[p\left(x,t\right)|p_{s}\left(x,t\right)\right]=-\dot{S}+\frac{1}{D}\frac{d}{dt}\left[\int dxVp-F_{s}\right]=-{\dot{S}}_{T}+\frac{1}{D}\left[\dot{W}-\frac{d}{dt}F_{s}\right]. \end{array}$$

One can see easily that equating Equation (42) to $\frac{1}{D}\left[\dot{F}-\dot{F_{s}}\right]$ (from Equation (40)) simply recovers $\dot{W}-\frac{d}{dt}F=D{\dot{S}}_{T}$ in Equation (35).

Finally, we obtain a differential form of Equation (41) by using ${\dot{W}}_{irr}=\dot{W}-\frac{d}{dt}F_{s}$ in Equation (42) as follows

(43) $${\dot{W}}_{irr}=D{\dot{S}}_{T}+D\frac{d}{dt}K\left[p\left(x,t\right)|p_{s}\left(x,t\right)\right].$$

### 4.4. Example

We consider v(t)=ut with a constant *u* so that $V=-\frac{\gamma }{2}{\left(x-ut\right)}^{2}$ in Equation (14). While the discussion below explicitly involves v(t), the results are general and valid for the limiting case v(t)=0. The case with v(t)=0 is an example where the forward and reversal protocols do not exist while a non-equilibrium stationary state does.

For f=−γ(x−ut), Equation (19) is simplified as follows

(44) $$\langle x\left(t\right)\rangle =x_{0}e^{-\gamma t}+ut-\frac{u}{\gamma }\left(1-e^{-\gamma t}\right).$$

For the non-equilibrium stationary state with fixed γ and *D*, $\beta_{s}=\frac{\gamma }{2D}$ is also constant ($\frac{d}{dt}F_{s}=0$). Therefore, we have

(45) $$p_{s}\left(x,t\right)=\sqrt{\frac{\beta_{s}}{\pi }}e^{-\beta_{s}{\left(x-ut\right)}^{2}}.$$

Then, we can find (see [29] for details)

(46) $$\begin{array}{ccc} K[p\left(x,t\right)|p_{s}\left(x,t\right)] & = & \frac{1}{2}\left[-1+\ln\left(\beta /\beta_{s}\right)\right]+\beta_{s}\left({\left(\langle x\rangle -ut\right)}^{2}+\frac{1}{2\beta }\right), \end{array}$$

(47) $$\begin{array}{ccc} D{\dot{S}}_{T} & = & \left[{\left(-\gamma \langle x_{0}\rangle e^{-\gamma t}+u\left(1-e^{-\gamma t}\right)\right)}^{2}+\frac{1}{2\beta }{\left(2\beta D-\gamma \right)}^{2}\right], \end{array}$$

(48) $$\begin{array}{ccc} \dot{Q} & = & {\left[-\gamma \langle x_{0}\rangle e^{-\gamma t}+u\left(1-e^{-\gamma t}\right)\right]}^{2}-\frac{\gamma }{2\beta }\left(2\beta D-\gamma \right), \end{array}$$

(49) $$\begin{array}{ccc} \dot{W} & = & -u\int dx\gamma \left(x-ut\right)p=-u\gamma \langle x-ut\rangle =u^{2}\left(1-e^{-\gamma t}\right), \end{array}$$

(50) $$\begin{array}{ccc} \frac{d}{dt}K\left[p\left(x,t\right)|p_{s}\left(x,t\right)\right] & = & -\frac{1}{D}\left[{\left(\partial_{t}\langle x\rangle \right)}^{2}+{\left(\partial_{t}\sigma \right)}^{2}-u\partial_{t}\langle x\rangle \right]=-{\dot{S}}_{T}+\frac{1}{D}\dot{W}. \end{array}$$

Here, we used Equations (23)–(26), 〈x〉 and β in Equations (44) and (18), respectively, and $\dot{W}=\langle -\gamma u\left(x-ut\right)\rangle =u\partial_{t}\langle x\rangle$.

It is worth looking at the two interesting limits of Equations (46)–(50). First, in the long time limit as t→∞, the following simpler relations are found:

(51) $$\begin{array}{ccc} & & \langle x\rangle \to u\left(t-\gamma^{-1}\right),\;2\beta \to \frac{\gamma }{D}=2\beta_{s},\\ & & D{\dot{S}}_{T}=\dot{Q}=\dot{W}={\left(\sigma \Gamma \right)}^{2}\to u^{2},\\ & & \dot{S}\to 0,\;\frac{d}{dt}K\left[p\left(x,t\right)|p_{s}\left(x,t\right)\right]\to 0. \end{array}$$

Equation (51) illustrates how the external force v(t)=ut≠0 keeps the system out of equilibrium even in the long time limit, with non-zero entropy production and dissipation. When there is no external force u=0, the system reaches equilibrium as t→∞, and all quantities in Equation (51) apart from β become zero.

The second is when the system is initially in equilibrium with $\beta \left(t=0\right)=\beta \left(t\to \infty \right)=\frac{\gamma }{2D}$ and $\langle x_{0}\rangle =0$ and evolve in time as it is driven out of equilibrium by u≠0. As *u* does not affect variance, $\beta \left(t\right)=\beta_{0}=\frac{\gamma }{2D}$ ($\partial_{t}\sigma =0$) and $\dot{S}=0$ for all time. In this case, we find

(52) $$\begin{array}{ccc} & & D{\dot{S}}_{T}=\dot{Q}=u^{2}{\left(1-e^{-\gamma t}\right)}^{2}={\left(\sigma \Gamma \right)}^{2},\;\dot{W}=u^{2}\left(1-e^{-\gamma t}\right),\\ & & DK\left[p\left(x,t\right)|p_{s}\left(x,t\right)\right]=\frac{u^{2}}{2\gamma }{\left(1-e^{-\gamma t}\right)}^{2},\\ & & D\frac{d}{dt}K\left[p\left(x,t\right)|p_{s}\left(x,t\right)\right]=u^{2}\left(1-e^{-\gamma t}\right)e^{-\gamma t}. \end{array}$$

Equation (52) shows that ${\dot{S}}_{T}$, $\dot{Q}$, $\Gamma^{2}$, $\dot{W}$, and K start with zero values at t=0 and monotonically increase to their asymptotic values as t→∞.

Finally, both cases considered above in Equations (51) and (52) have $\partial_{t}\sigma =0$ and thus recover Equation (30):

(53) $$\Gamma^{2}=\frac{D{\dot{S}}_{T}}{\sigma^{2}}=\frac{\dot{Q}}{\sigma^{2}}.$$

## 5. Inequalities

Section 4 utilized the average (first moment) of a variable (e.g., 〈V〉) and the average of its first time derivative ($\langle \partial_{t}V\rangle =\dot{W}$) while the work $W=\int dt\;\dot{W}$ is defined by the time integral of $\dot{W}=\langle \partial_{t}V\rangle$ in Equation (33). This section aims to show that the rates at which average quantities vary with time are bounded by fluctuations and Γ. Since the average and time derivatives do not commute, we pay particular attention to when the average is taken.

To this end, let us first define the microscopic free energy μ=V+Dlnp (called the chemical potential energy in [113]). In terms of μ, we have $J=-p\partial_{x}\mu$ and 〈μ〉=U−DS=F. On the other hand,

(54) $$\begin{array}{ccc} \partial_{t}\mu =\partial_{t}V+D\frac{\dot{p}}{p}, & & \langle \partial_{t}\mu \rangle =\langle \partial_{t}V\rangle =\dot{W}. \end{array}$$

$\langle \partial_{t}\mu \rangle =\dot{W}$ means that the average rate of change in the microscopic free is the power. From Equation (54), it follows

(55) $$\langle {\left(\partial_{t}\mu -\partial_{t}V\right)}^{2}\rangle =D^{2}\int dx\frac{{\dot{p}}^{2}}{p}=D^{2}\Gamma^{2}.$$

Equation (55) establishes the relation between the microscopic free energy and Γ.

Next, we calculate the time-derivative of F

(56) $$\begin{array}{ccc} \frac{d}{dt}F & = & \frac{d}{dt}\langle \mu \rangle =\langle \dot{\mu }\rangle +\int dx\mu \dot{p}=\dot{W}+\int dx\mu \dot{p}. \end{array}$$

Using $\frac{d}{dt}F=\dot{W}-D{\dot{S}}_{T}$ in Equation (56) gives ${\dot{S}}_{T}$ in terms of μ as

(57) $$\begin{array}{ccc} \dot{W}-\frac{d}{dt}F=D\dot{S_{T}} & = & -\int dx\mu \dot{p}. \end{array}$$

Equation (57) is to be used in Section 5.1 for linking ${\dot{S}}_{T}$ to Γ through an inequality.

### 5.1. General Inequality Relations

We now use $\int dx\dot{p}=0$, $\int dx\dot{p}\langle A\rangle =\langle A\rangle \int dx\dot{p}=0$ for any A=A(x,t) and apply the Schwartz inequality $|\int^{t}dt_{1}A\left(t_{1}\right)B\left(t_{1}\right)|\leq {\left[\int^{t}dt_{1}A^{2}\left(t_{1}\right)\right]}^{\frac{1}{2}}{\left[\int^{t}dt_{1}B^{2}\left(t_{1}\right)\right]}^{\frac{1}{2}}$ to Equations (21), (34) and (57) to obtain

(58) $$\begin{array}{ccc} |\dot{S}| & = & |\int dx\dot{p}\ln p|\leq \Gamma {\left[\int dxp{\left(\delta \ln p\right)}^{2}\right]}^{1/2}, \end{array}$$

(59) $$\begin{array}{ccc} |\dot{Q}| & = & |\int dxV\dot{p}|\leq \Gamma {\left[\int dxp{\left(\delta V\right)}^{2}\right]}^{1/2}, \end{array}$$

(60) $$\begin{array}{ccc} D{\dot{S}}_{T} & = & \left|\int dx\mu \dot{p}\right|\leq \Gamma {\left[\int dxp{\left(\delta \mu \right)}^{2}\right]}^{1/2}. \end{array}$$

Equation (60) (Equation (59)) establishes the inequality between entropy production rate (heat flux) and the product of the RMS fluctuations of the microscopic free energy (potential energy) and Γ. Since δμ=δV+D(δlnp), we have

(61) $$\begin{array}{ccc} \langle {\left(\delta \mu \right)}^{2}\rangle & = & \langle {\left(\delta V\right)}^{2}\rangle +D^{2}\langle {\left(\delta \ln p\right)}^{2}\rangle +2D\langle \delta V\delta \ln p\rangle . \end{array}$$

These relations are to be used in Section 5.2 below.

### 5.2. Applications to the Non-Autonomous O-U Process

For a linear O-U process with $V=\frac{\gamma }{2}{\left(x-v\left(t\right)\right)}^{2}$ and f=−γ(x−v(t)) in Equation (14), we use $\langle {\left(\delta x\right)}^{2}\rangle =\frac{1}{2\beta }$, $\langle {\left(\delta x\right)}^{4}\rangle =3\langle {\left(\delta x\right)}^{2}\rangle =\frac{3}{4\beta^{2}}$ and $\partial_{t}\langle x\rangle =-\gamma \langle x-v\left(t\right)\rangle$ to show

(62) $$\begin{array}{ccc} \delta V & = & -\partial_{t}\langle x\rangle \delta x+\frac{\gamma }{2}\left[{\left(\delta x\right)}^{2}-\frac{1}{2\beta }\right]\\ \langle {\left(\delta V\right)}^{2}\rangle & = & {\left(\partial_{t}\langle x\rangle \right)}^{2}\sigma^{2}+\gamma^{2}\frac{\sigma^{4}}{2},\\ \delta \ln p & = & \frac{1}{2}-\beta {\left(\delta x\right)}^{2},\\ \langle {\left(\delta \ln p\right)}^{2}\rangle & = & \frac{1}{2},\\ \langle (\delta \ln p)(\delta V)\rangle & = & -\frac{\gamma }{4\beta }. \end{array}$$

Using Equations (61)–(62) in Equations (58)–(60) together with $2D\beta -\gamma =-\frac{\partial_{t}\beta }{2\beta }$ and $\frac{\partial_{t}\beta }{\beta }=-2\frac{\partial_{t}\sigma }{\sigma }$ leads to

(63) $$\begin{array}{ccc} |\dot{S}| & \leq & \frac{1}{\sqrt{2}}\Gamma ,\\ |\dot{Q}| & \leq & \Gamma \sigma {\left[{\left(\partial_{t}\langle x\rangle \right)}^{2}+\frac{\gamma^{2}\sigma^{2}}{2}\right]}^{\frac{1}{2}},\\ D{\dot{S}}_{T} & \leq & \Gamma \sigma {\left[{\left(\partial_{t}\langle x\rangle \right)}^{2}+\frac{1}{2}{\left(\partial_{t}\sigma \right)}^{2}\right]}^{\frac{1}{2}}. \end{array}$$

Finally, it is useful to examine the extreme cases of Equation (63). First, when $\partial_{t}\sigma =0$, Equation (63) holds as an equality as $D{\dot{S}}_{T}={\left(\partial_{t}\langle x\rangle \right)}^{2}=\sigma^{2}\Gamma^{2}$ (see Equation (27)), recovering Equation (28) with $\partial_{t}\sigma =0$. Second, when $\partial_{t}\langle x\rangle =0$, Equation (63) again holds as an equality since $D{\dot{S}}_{T}={\left(\partial_{t}\sigma \right)}^{2}$ and $\Gamma \sqrt{\frac{1}{2}\sigma^{2}{\left(\partial_{t}\sigma \right)}^{2}}={\left(\partial_{t}\sigma \right)}^{2}$.

## 6. Geodesics, Control and Hyperbolic Geometry

The section aims to discuss geodesics in information geometry and its implications for self-organization and control. To illustrate the key concepts, we utilize an analytically solvable, generalized O-U process given by

(64) $$\frac{dx}{dt}=-\gamma \left(t\right)\left[x-v\left(t\right)\right]+\xi ,$$

where γ(t)>0 is a damping constant; v(t) is a deterministic force which determines the time evolution of the mean value of *x*; ξ is a short (delta)-correlated noise with the time-dependent amplitude D(t) in general, satisfying Equation (15).

For the initial condition in Equation (16), the mean value 〈x〉≡y(t) and β(t) are given by

(65) $$\begin{array}{ccc} y(t) & = & \langle x\rangle =x_{0}e^{-\int_{0}^{t}dt_{1}\gamma \left(t_{1}\right)dt_{1}}+\int_{0}^{t}dt_{1}e^{-\int_{0}^{t}dt_{1}\left[\gamma \left(t_{1}\right)-\gamma \left(t\right)\right]dt_{1}}\;\gamma \left(t_{1}\right)\;f\left(t_{1}\right)\;, \end{array}$$

(66) $$\begin{array}{ccc} \frac{1}{2\beta (t)} & = & \langle {\left(x-\langle x\rangle \right)}^{2}\rangle =\frac{e^{-2\int_{0}^{t}dt_{1}\gamma \left(t_{1}\right)dt_{1}}}{2\beta_{0}}+\int_{0}^{t}dt_{1}e^{-2\int_{0}^{t}dt_{1}\left[\gamma \left(t_{1}\right)-\gamma \left(t\right)\right]dt_{1}}\;2D\left(t_{1}\right), \end{array}$$

where $x_{0}=\langle x\left(t=0\right)\rangle$.

### 6.1. Geodesics–Shortest-Distance Path

A geodesics between the two spatial locations is a unique path with the shortest distance. A similar concept can be applied to information geometry to define a unique evolution path between the two given PDFs, say, $p(x,t_{1})$ and $p(x,t_{2})$ in the statistical space. The Wootters’ distance in quantum mechanics in Equation (4) is such an example. For time-varying stochastic processes, there is an infinite number of different trajectories between the two PDFs at different times. The key question that we address in this section is how to find an exact time evolution of p(x,t) when initial and final PDFs [15] are given. This is a much more difficult problem than finding a minimum distance between two PDFs (like the Wootter’s distance). In the following, we sketch some main steps needed for finding such a unique evolution path (the so-called geodesics) between given initial and final PDFs by minimizing L (see [15] for detailed steps).

For the O-U process in Equation (64), a geodesic solution does not exist for constant γ, v(t) and *D*. Thus, finding a geodesic solution boils down to determing suitable functions of γ(t), v(t) or D(t) [15]. To be specific, let $p(x,t_{0})$ and $p(x,t_{F})$, respectively, be the PDFs at the time $t=t_{0}$ and $t_{F}$ ($>t_{0}$) and find a geodesic solution by minimizing $L\left(t\right)=\int_{t_{0}}^{t_{F}}dt^{\prime }\;\Gamma \left(t^{\prime }\right)$. The latter is equivalent to minimizing $\int_{t_{0}}^{t_{F}}dt^{\prime }\;E\left(t^{\prime }\right)$ and to keeping Γ constant. (This geodesics is also called an optimal path (e.g., see [107]).) We rewrite E in Equation (27) for the O-U process in terms of y=〈x〉

(67) $$E=\frac{1}{2\beta^{2}}{\left(\frac{d\beta }{dt}\right)}^{2}+2\beta {\left(\frac{dy}{dt}\right)}^{2}.$$

The Euler-Lagrange equation

(68) $$\begin{array}{c} 0=\frac{dE}{d\beta }-\frac{d}{dt}\frac{dE}{d\dot{\beta }},\;\;\;0=\frac{dE}{dy}-\frac{d}{dt}\frac{dE}{d\dot{y}}\; \end{array}$$

($\dot{\beta }=\frac{d\beta }{dt}$ and $\dot{y}=\frac{dy}{dt}$) then gives us

(69) $$\begin{array}{ccc} & & \frac{d^{2}\beta }{dt^{2}}-\frac{1}{\beta }{\left(\frac{d\beta }{dt}\right)}^{2}-2\beta^{2}{\left(\frac{dy}{dt}\right)}^{2}=0\;, \end{array}$$

(70) $$\begin{array}{ccc} & & \frac{d}{dt}\left[\beta \frac{dy}{dt}\right]=0\to \beta \frac{dy}{dt}=c\;, \end{array}$$

where *c* is constant. An alternative method of obtaining Equations (69) and (70) is provided in Appendix C. The following equations are obtained from Equations (69) and (70) [15]

(71) $$\begin{array}{ccc} {\left(\frac{d\beta }{dt}\right)}^{2} & = & -4c^{2}\beta +\alpha \beta^{2}\;, \end{array}$$

(72) $$\begin{array}{ccc} \Gamma^{2} & = & \frac{1}{2\beta^{2}}{\dot{\beta }}^{2}+2c^{2}\beta =\frac{\alpha }{2}\;, \end{array}$$

where α is another (integration) constant. General solutions to Equations (70) and (71) for c≠0 were found in terms of hyperbolic functions as [15]

(73) $$\begin{array}{ccc} \beta \left(t\right)=\frac{4c^{2}}{\alpha }\cosh^{2}\left[\frac{1}{2}\sqrt{\alpha }\left(t-A\right)\right], & & y\left(t\right)=\frac{\sqrt{\alpha }}{2c}\tanh\left[\frac{1}{2}\sqrt{\alpha }\left(t-A\right)\right]-\frac{\sqrt{\alpha }}{2c}+B, \end{array}$$

where *A* and *B* are constant.

**Figure 4:**
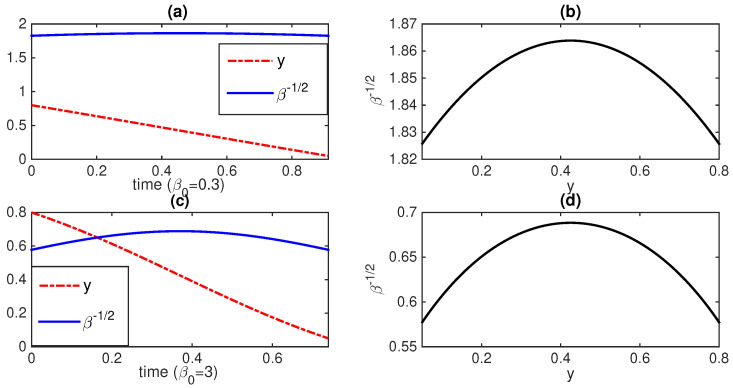
*y* and $\beta^{-1/2}$ against time for $\beta_{0}=0.3$ and 3 in (**a**,**c**), respectively; the corresponding geodesic circular segments in the $\left(y,\beta^{-1/2}\right)$ upper half-plane in (**b**,**d**), respectively. In both cases, $y_{0}=\frac{5}{6}$ and $y_{F}=\frac{1}{30}$. (Figure 3 in [15]).

Equation (73) can be rewritten using $\sigma ={\left(2\beta \right)}^{-\frac{1}{2}}$ and $z=\frac{y}{\sqrt{2}}$ as follows

(74) $${\left(z-z_{c}\right)}^{2}+\sigma^{2}=R^{2},\;\;z_{c}=\frac{B}{\sqrt{2}}-sR,\;\;R=\frac{\Gamma }{2c},$$

where *s* denotes the sign of *c* so that s=1 when c>0 while s=−1 when c<0. Equation (74) is an equation of a circle for the variables *z* and σ with the radius *R* and the center $z_{c}$, defined in the upper-half plane where σ≥0. Thus, geodesic motions occur along the portions of a circle as long as c≠0 (as can be seen in Figure 4). A geodesic moves on a circle with a larger radius for a larger information rate Γ and speed and vice versus. This manifests the hyperbolic geometry in the upper half Poincaré model [13,117] where the half-plane represents *z* and σ≠0 (see also Appendix D). The constants c,α,A, and *B* determine the coordinate of the center and the radius of the circle *R*. These constants should be fixed by the fixed conditions at the initial t=0 and final time $t_{F}$.

Having found the most general form of the geodesic solution for y(t) and β, the next steps require finding the values of constant values c,α,A,B to satisfy the boundary conditions at $t=t_{0}$ and $t_{F}$, and then finding appropriate γ(t), D(t), and v(t) that ensure the geodesic solutions. This means the O-U process should be controlled by γ(t), D(t) and v(t) to ensure a geodesic solution.

Figure 4 shows an example of a geodesic solution in the upper half-plane *y* and $\beta^{-1/2}$ when γ(t)=1 is constant while D(t) and v(t) are time-dependent. The boundary conditions are chosen as $y\left(t_{0}\right)=y_{0}=\frac{5}{6}$ and $y\left(t_{F}\right)=y_{F}=\frac{1}{30}$ in all panels (a)–(d). $\beta \left(t_{0}\right)=\beta_{0}=\beta \left(t_{F}\right)=\beta_{F}=0.3$ in panels (a) and (b) while $\beta_{0}=\beta_{F}=3$ in panels (c) and (d). Interestingly, circular-shape phase-portraits are seen in panels (b) and (d), reflecting hyperbolic geometry noted above (see also Appendix D) [13,117]. The speed at which the geodesic motion takes place in the phase portrait is determined by the constant value of $\Gamma =\sqrt{\frac{\alpha }{2}}$ (i.e., the larger α, the faster time evolution).

**Figure 5:**
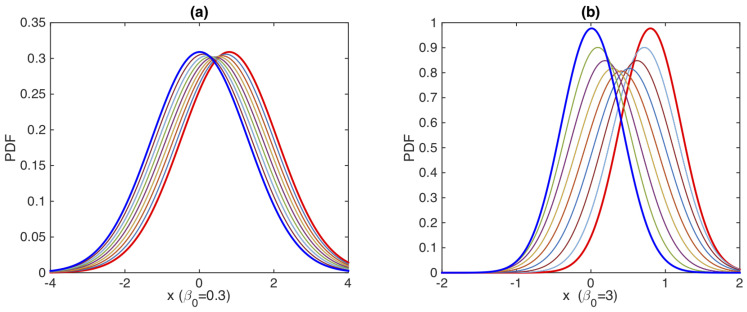
Time evolution of PDFs against *x*: (**a**) $\beta_{0}=0.3$ corresponding to Figure 4a,b; (**b**) $\beta_{0}=3$ corresponding to Figure 4c,d. In both cases, $y_{0}=\frac{5}{6}$ and $y_{F}=\frac{1}{30}$. The initial and final PDFs are shown by thick red and blue lines, respectively. (Figure 4a,b in [15]).

Figure 5a,b are the corresponding PDF snapshots at different times (shown in different colors), demonstrating how the PDF evolves from the initial PDF in red to the final PDF in blue. In both cases, it is prominent that the PDF width ($\propto \beta^{-1/2}$) initially broadens and then becomes narrower.

### 6.2. Comments on Self-Organization and Control

Self-organization (also called homeostasis) is the novel phenomena where order spontaneously emerges out of disorder and is maintained by different feedbacks in complex systems [45,52,53,118,119,120,121,122,123]. The extremum principles of thermodynamics such as the minimum entropy production (e.g., [119,121]) or maximum entropy entropy production (e.g., [122,123]) have been proposed by considering a steady state or an instant time in different problems.

However, far from equilibrium, self-organization can be a time-varying non-equilibrium process involving perpetual or large fluctuations (e.g., see [52,53,54]). In this case, the extreme of entropy production should be on accumulative entropy production over time rather than at one instant time nor in a steady state. That is, we should consider the time-integral of the entropy production ${\dot{S}}_{T}$, or equivalently, the time-integral of $\sqrt{{\dot{S}}_{T}}$. As seen from Equations (24) and (53), for a linear O-U process with a constant variance, there is an exact proportionality between $\sqrt{{\dot{S}}_{T}}$ and Γ. In this case, the extreme of $L\left(t\right)=\int^{t}dt_{1}\Gamma \left(t_{1}\right)$ would be the same as the extreme of $\int^{t}dt_{1}\sqrt{{\dot{S}}_{T}}$. However, as noted previously, $\Gamma \propto \sqrt{{\dot{S}}_{T}}$ does not hold in general (e.g., see Equation (28)).

With these comments, we now look at the implications of a geodesic for self-organization, in particular, in biosystems. For the very existence and optimal functions of a living organiss, it is critical to minimize the dispersion of its physical states and to maintain its states within certain bounds upon changing conditions [124]. How fast its state changes in time can be quantified by the surprise rate $\partial_{t}\left[\ln\left(p(x,t\right)\right]$. Since $\int dxp\left(x,t\right)\partial_{t}\ln\left(p(x,t)\right)=0$, we use its RMS value $\sqrt{\langle {\left(\partial_{t}\ln p\right)}^{2}\rangle }=\Gamma$ (see Equation (9)) and realize that the total change over a finite time interval $\left[t_{0},t_{F}\right]$ is nothing more than $L_{(}t)=\int_{t_{0}}^{t_{F}}dt_{1}\;\Gamma \left(t_{1}\right)$. Thus, minimizing the accumulative/time-integral of the RMS surprise rate is equivalent to minimizing L. Envisioning surprise rate as biological cost associated with changes (e.g., needed in updating the future prediction based on the current state [124,125]), we can then interpret L as an accumulative biological cost. Thus, geodesic would be an optimal path that minimizes such an accumulative biological cost.

Ref [15] addressed how to utilize this idea to control populations (tumors). Specifically, the results in Section 6.1 were applied to a nonlinear stochastic growth model (obtained by a nonlinear change of variables of the O-U process), and the geodesic solution in Equation (73) was used to find the optimal protocols v(t) and D(t) in reducing a large-size tumor to a smaller one. Here, in this problem, D(t) represents the heterogeneity of tumor cells (e.g., larger *D* for metastatic tumor) that can be controlled by gene reprogramming while v(t) models the effect of a drug or radiation that reduces the mean tumor population/size.

## 7. Discussions and Conclusions

There has been a growing interest in information geometry from theoretical and practical considerations. This paper discussed some recent developments in information geometric theory, focusing on time-dependent *dynamic* aspects of non-equilibrium processes (e.g., time-varying mean value, time-varying variance, or temperature) and their thermodynamic and physical/biological implications.

In Section 2 and Section 3, by utilizing a Langevin model of an over-damped stochastic process x(t), we highlighted the importance of a *path-dependent* distance L in describing time-varying processes. In Section 4 and Section 5, we elucidated the thermodynamic meanings of the relative entropy and the information rate Γ by relating them to the entropy production rate (${\dot{S}}_{T}$), $\dot{S}$, heat flux ($Q=D{\dot{S}}_{m}$), dissipated work (${\dot{W}}_{D}$), etc., and demonstrated the role of Γ in determining bounds (or speed limit) on thermodynamical quantities.

Specifically, in the O-U process, we showed the exact relation $\Gamma =\sqrt{\frac{D}{\sigma^{2}}{\dot{S}}_{T}+{\dot{S}}^{2}}$ (Equation (28)), which is simplified as $\sigma \Gamma =\sqrt{D{\dot{S}}_{T}}$ when $\partial_{t}\sigma =0$ ($\sigma =\sqrt{\langle {\left(\delta x\right)}^{2}\rangle }$ is the standard deviation of *x*). Finally, Section 6 discussed geodesic and its implication for self-organization as well as the underlying hyperbolic geometry. It remains future works to explore the link between Γ and the entropy production rate in other (e.g., nonlinear) systems consisting of three or more interacting components or data from self-organizing systems (e.g., normal brain).

## Data Availability

Data are available from the author.

## Appendix A

In this Appendix, we show the invariance of Equation (9) when *x* changes as y=F(x). Using the conservation of the probability, we then have

(A1) $$p\left(y,t\right)=p\left(x,t\right)\left|\frac{dx}{dy}\right|=p\left(x,t\right){\left|\frac{dF(x)}{dx}\right|}^{-1},$$

Since $\frac{dF(x)}{dx}$ is independent of time *t*, it follows that $\partial_{t}p\left(y,t\right)=\left[\partial_{t}p\left(x,t\right)\right]{\left|\frac{dF(x)}{dx}\right|}^{-1}$. Using this and $dy=dx\left|\frac{dF(x)}{dx}\right|$, we have

(A2) $$\int dy\frac{1}{p(y,t)}{\left[\frac{\partial p(y,t)}{\partial t}\right]}^{2}=\int dx\frac{1}{p(x,t)}{\left[\frac{\partial p(x,t)}{\partial t}\right]}^{2}.$$

This shows that p(x,t) and p(y,t) give the same $\Gamma^{2}\left(t\right)$.

## Appendix B. The Coupled O-U Process

The coupled O-U process for Figure 3 in Section 3.1 is governed by the Fokker-Planck equation [22]

(A3) $$\frac{\partial P_{1}}{\partial t}=\frac{\partial }{\partial x}\left[\gamma_{1}\left(x-\mu \right)P_{1}\right]+D\frac{\partial^{2}P_{1}}{\partial x^{2}}-f_{0}P_{1}+g_{0}P_{2},$$

(A4) $$\frac{\partial P_{2}}{\partial t}=\frac{\partial }{\partial x}\left[\gamma_{2}\left(x-\mu \right)P_{2}\right]+D\frac{\partial^{2}P_{2}}{\partial x^{2}}+f_{0}P_{1}-g_{0}P_{2}.$$

Here, *D* is the strength of a short-correlated Gaussian noise given by Equation (15). These equations are the coupled O-U processes with the coupling constants $f_{0}$ and $g_{0}$ through the Dichotomous noise [110,111].

For simplicity, we use $\gamma_{1}=\gamma_{2}=\gamma$ and $f_{0}=g_{0}=\epsilon$ and the following initial conditions

(A5) $$\begin{array}{ccc} P_{1}\left(x,0\right) & = & \frac{1}{2}\sqrt{\frac{\beta_{10}}{\pi }}\exp\left[-\beta_{10}x^{2}\right], \end{array}$$

(A6) $$\begin{array}{ccc} P_{2}\left(x,0\right) & = & \frac{1}{2}\sqrt{\frac{\beta_{20}}{\pi }}\exp\left[-\beta_{20}{\left(x-x_{0}\right)}^{2}\right]. \end{array}$$

The solutions are given by

(A7) $$\begin{array}{ccc} P_{1}\left(x,t\right) & = & \frac{1}{4}\left[\sqrt{\frac{\beta_{1}}{\pi }}\left(1+e^{-2\epsilon t}\right)e^{-\beta_{1}x^{2}}+\sqrt{\frac{\beta_{2}}{\pi }}\left(1-e^{-2\epsilon t}\right)e^{-\beta_{2}{\left(x-e^{-\gamma t}x_{0}\right)}^{2}}\right], \end{array}$$

(A8) $$\begin{array}{ccc} P_{2}\left(x,t\right) & = & \frac{1}{4}\left[\sqrt{\frac{\beta_{1}}{\pi }}\left(1-e^{-2\epsilon t}\right)e^{-\beta_{1}x^{2}}+\sqrt{\frac{\beta_{2}}{\pi }}\left(1+e^{-2\epsilon t}\right)e^{-\beta_{2}{\left(x-e^{-\gamma t}x_{0}\right)}^{2}}\right], \end{array}$$

where

(A9) $$\frac{1}{2\beta_{m}}=\frac{e^{-2\gamma t}}{2\beta_{m0}}+\frac{D}{\gamma }\left(1-e^{-2\gamma t}\right),$$

for m=1,2. In the limit of t→∞, $P_{1}$ and $P_{2}$ in Equations (A7) and (A8) approach the same equilibrium distribution

(A10) $$\begin{array}{c} P_{m}\left(x,t\right)=\frac{1}{2}\sqrt{\frac{\beta_{m}\left(t\to \infty \right)}{\pi }}e^{-\beta_{m}\left(t\to \infty \right)x^{2}}, \end{array}$$

where $\beta_{m}\left(t\to \infty \right)=\frac{\gamma }{2D}$. We note that the total PDF $P=P_{1}+P_{2}$ satisfies the single O-U process where the initial PDF is given by the sum of Equations (A5) and (A6).

Figure 3 is shown for the fixed parameter values γ=0.1, D=1 and ϵ=0.5, $\beta_{20}=\beta_{10}=0.05=\beta \left(t\to \infty \right)=\frac{\gamma }{2D}=0.05$. Different values of the initial mean position $x_{0}$ of $P_{2}$ are used to examine how metrics depend on $x_{0}$. As noted in Section 2.2, $P_{1}$ at t=0 is chosen to be the same as the final equilibrium state which has the zero mean value and inverse temperature $\beta_{10}=0.05$.

## Appendix C. Curved Geometry: The Christoffel and Ricci-Curvature Tensors

A geodesic solution in Section 6.1 can also be found by solving the geodesic equation in general relativity (e.g., [31,107]). To this end, we let the two parameters be $\lambda^{1}=\langle x\rangle =y$ and $\lambda^{2}=\beta$ and express Equation (A11) in terms of the metric tensor $g_{ij}$ as follows (see also Equation (10))

(A11) $$E=\sum_{ij}\frac{d\lambda^{i}}{dt}g_{ij}\frac{d\lambda^{j}}{dt},$$

where

(A12) $$g_{ij}=\left(\begin{array}{cc} \frac{1}{2\beta^{2}} & 0\\ 0 & 2\beta \end{array}\right),\;\;\;\;\;\;\;\;\lambda^{i}=\left(\begin{array}{c} \beta \\ y \end{array}\right)\;.$$

Note that while $g_{ij}$ is diagonal, the 1-st diagonal component depends on β (the second parameter). That is, $g_{ii}$ is not independent of *j*-th parameter for j≠i in general. From Equation (A12), we can find non-zero components of connection tensor $\Gamma_{ijk}=\frac{1}{2}\left[\partial_{i}g_{jk}+\partial_{j}g_{ik}-\partial_{k}g_{ij}\right]$ ($\Gamma_{jk}^{i}=g_{im}\Gamma_{jkm}$)

(A13) $$\Gamma_{11}^{1}=-\frac{1}{\beta },\Gamma_{22}^{1}=-2\beta^{2},\Gamma_{12}^{2}=\Gamma_{21}^{2}=\frac{1}{2\beta }\;.$$

A geodesic equation $\frac{d^{2}\lambda^{i}}{dt^{2}}+\Gamma_{mk}^{i}\frac{d\lambda^{m}}{dt}\frac{d\lambda^{k}}{dt}=0$ in terms of the Christoffel tensors becomes

(A14) $$\begin{array}{ccc} & & \ddot{\beta }+\Gamma_{11}^{1}{\dot{\beta }}^{2}+\Gamma_{22}^{1}{\dot{y}}^{2}=0,\; \end{array}$$

(A15) $$\begin{array}{ccc} & & \ddot{y}+\Gamma_{12}^{2}\dot{\beta }\dot{y}+\Gamma_{21}^{2}\dot{\beta }\dot{y}=0.\; \end{array}$$

Equations (A13)–(A15) give Equation (70). Note that if $g_{ii}$ is independent of the $\lambda^{j}$ (j≠i) for all *i* and *j*, the Christoffel tensors have non-zero values only for $\Gamma_{ii}^{i}$, leading to a much simpler geodesic solution (e.g., see [31]).

Finally, to appreciate the curved geometry associated with this geodesic solution, we proceed to calculate the Riemann curvature tensor $R_{kmn}^{i}=\partial_{m}\Gamma_{nk}^{i}+\Gamma_{mp}^{i}\Gamma_{nk}^{p}-\partial_{n}\Gamma_{mk}^{i}-\Gamma_{np}^{i}\Gamma_{mk}^{p}$ and the Ricci tensor $R_{ij}=R_{ikj}^{k}$ from Equation (A13) and find the following non-zero components [15]

(A16) $$R_{212}^{1}=-R_{221}^{1}=-\beta ,\;\;\;R_{112}^{2}=-R_{121}^{2}=\frac{1}{4\beta^{2}}.$$

Non-zero curvature tensors represent that the metric space is curved with a finite curvature. Specifically, we find the Ricci tensor $R_{ij}=R_{ikj}^{k}$ and curvature *R*:

(A17) $$\begin{array}{c} R_{11}=-\frac{1}{4\beta^{2}},\;\;R_{22}=-\beta ,\;\;\;R_{12}=R_{21}=0,\;\; \end{array}$$

(A18) $$\begin{array}{c} R=g_{ij}R_{ij}=-1\; \end{array}$$

The negative curvature is typical of hyperbolic geometry. Finally, using R=−1, we calculate the Einstein field equation

(A19) $$G_{ij}=R_{ij}-\frac{1}{2}R\;g_{ij}=\left(\begin{array}{cc} -\frac{1}{4\beta^{2}} & 0\\ 0 & -\beta \end{array}\right)+\frac{1}{2}\left(\begin{array}{cc} \frac{1}{2\beta^{2}} & 0\\ 0 & 2\beta \end{array}\right)=0\;.$$

Since $G_{ij}=8\pi T_{ij}$ where $T_{ij}$ is the stress-energy tensor, we see that $T_{ij}=0$ for this problem.

## Appendix D. Hyperbolic Geometry

The Hyperbolic geometry in the upper-half plane [13,117] becomes more obvious when Equation (A12) is expressed in terms of the two parameters 〈x(t)〉 and σ(t) where *x* and *y* axes represent 〈x(t)〉 and σ(t) with the metric tensor

(A20) $$g_{ij}^{H}=\left(\begin{array}{cc} \frac{2}{\sigma^{2}} & 0\\ 0 & \frac{1}{\sigma^{2}} \end{array}\right).$$
